# Correlation Between Serum Magnesium Level and Acute Exacerbation in Patients With Chronic Obstructive Pulmonary Disease (COPD)

**DOI:** 10.7759/cureus.26229

**Published:** 2022-06-23

**Authors:** Sanket Makwana, Archit Patel, Maganlal Sonagara

**Affiliations:** 1 General Medicine, C. U. Shah Medical College, Surendranagar, IND

**Keywords:** global initiative for obstructive lung disease (gold), normomagnesemia, hypomagnesemia, serum magnesium, chronic obstructive pulmonary disease (copd)

## Abstract

Introduction

Although chronic obstructive pulmonary disease (COPD) is preventable and treatable, it is a major public health problem. The mortality risks are higher in patients with exacerbations. Magnesium (Mg) is crucial in numerous physiological processes, including membrane stabilization. However, incomplete information is available regarding the effect of magnesium on the frequency of acute exacerbation of COPD.

Objectives

To determine the serum magnesium level in COPD patients and its correlation with acute exacerbation of COPD.

Materials and methods

This cross-sectional study included 100 patients diagnosed with acute exacerbation of COPD. The serum magnesium level was measured in all patients with acute exacerbation of COPD at admission. Serum Mg level <1.7 mg/dl was considered hypomagnesemia. The correlation between serum magnesium level and duration of hospital stay and patient outcome was studied.

Results

In the present study, hypomagnesemia was reported in 57% of patients with acute exacerbation of COPD. The duration of hospital stay (more than seven days) among hypomagnesemia (80.7%) patients was significantly higher than that of the normomagnesemia patients (55.8%). Mortality in patients with hypomagnesemia was higher than in patients with normomagnesemia, although not statistically significant.

Conclusion

Hypomagnesaemia is a common finding in acute exacerbation of COPD. The level of magnesium found is related to the length of hospital stay, but it is not related to mortality among patients with acute exacerbation of COPD. Further studies with larger sample sizes and extended follow-up periods are required to validate the results.

## Introduction

Although chronic obstructive pulmonary disease (COPD) is preventable and treatable, it is one major public health problem. In the United States, COPD is the fourth most common cause of mortality, and its prevalence is gradually increasing worldwide [1]. It is expected that around 250 million people are already suffering from COPD [1].

Global initiatives of chronic obstructive pulmonary disease (GOLD) defined COPD as a common, preventable, and treatable disease that is characterized by persistent respiratory symptoms and airflow limitation due to airway and/or alveolar abnormalities, usually caused by significant exposure to the noxious particles or gases [2]. In a developing country like India, COPD’s most common risk factors include smoking and non-smoking-related factors such as occupational exposure to dust and fumes, pollen grains, crop dust, exposure to biomass fuels while cooking, lower socioeconomic status, and overcrowding [3-4].

The frequency and severity of COPD exacerbations have increased in the last few decades [5]. The hospital admission rate due to acute exacerbations of COPD is also progressively raised, especially in the winter season. The injudicious use of corticosteroids and bronchodilators, irrational uses of antibiotics, low pre-treatment FEV1 (forced expiratory volume in the first second), history of more than three episodes of exacerbation in the previous two years, and comorbid or immunocompromised state are the common risk factors for exacerbation relapses of COPD [6-9]. The risk of death is higher in a group of patients requiring frequent hospitalization or having a history of frequent episodes of acute exacerbation than in stable COPD patients [10].

An exacerbation of COPD is defined as a persistent worsening of a patient's symptoms from its baseline, which is abrupt in onset and beyond its daily variation and may require a change in medicines or hospitalization for symptoms [11]. Several large COPD studies have defined exacerbations of COPD as "a worsening of respiratory symptoms, which required treatment with oral corticosteroids or antibiotics, or both" [12].

Magnesium plays a vital role in various intracellular physiological processes, including membrane stabilization [13]. Magnesium has mainly bronchodilator properties in the airways of COPD. Inhibitory effect on bronchial smooth muscle contraction by calcium, inhibitory effect on acetylcholine release from cholinergic nerve terminal, and histamine release from mast cells are different mechanisms for bronchodilator activities of magnesium [14-15]. It has been observed that a very low dietary intake of magnesium is a risk factor for developing asthma and COPD [16].

However, incomplete information is available concerning the effect of magnesium on the frequency of acute exacerbation of COPD and the role of magnesium in reducing hospital stays and mortality related to the exacerbations. Therefore, the present study was conducted to determine the serum magnesium level in COPD patients during acute exacerbation and its correlation with acute exacerbation of COPD.

## Materials and methods

Study design and ethical approval

The cross-sectional observational study was conducted in the Department of Medicine, C. U. Shah Medical College, Surendranagar, a tertiary care center in the Saurashtra region of Gujarat, from January 2019 to June 2020. We did a pilot study and found our prevalence of hypomagnesemia was 7%. We calculated the sample size using the formula, sample size (N) = Z2*PQ/e2, where z is a confidence level of 95% (1.96), p means prevalence, q means 100-p, and e means allowable error (5%). Based on this, we have included 100 patients diagnosed with acute exacerbation of COPD presenting at the outpatient department (OPD) or the emergency department (ED) of our institute based on inclusion and exclusion criteria. The ethical approval was taken from the institutional ethical committee with reference no. CUSMC/IEC(HR)/PUB-14/2022/Final Approval/109/2022.

Inclusion criteria

All diagnosed cases of COPD aged more than 40 years presented with acute exacerbation of COPD in the OPD or ED.

Exclusion criteria

We have excluded those patients who required intubation or noninvasive ventilation (NIV), were not able to perform spirometry, have evidence of pneumothorax, hypotension, any other serious medical condition like a cerebrovascular accident, ischemic heart disease, arteriosclerotic disease, chronic alcoholism, gastrointestinal surgery or taking regular medications like H2 blocker, proton pump inhibitors, thiazide, and digoxin at the time of admission.

Methodology

Before enrolling in the study, informed written consent was taken from the study participant. A preformed semi-structured proforma was used to collect basic demographic information and record each study subject’s detail. As there is no specific biomarker for COPD, diagnosis of acute exacerbation of COPD relies on symptoms and clinical assessment for acute exacerbations of COPD. As per the GOLD criteria, COPD exacerbations are acute worsening of respiratory symptoms that are acute in onset and result in additional therapy [17]. The serum magnesium level was measured in all patients at admission by colorimetry using the Merylizer AutoQuant 200 Excelus (Meril Life, Gujarat, India) fully automated biochemistry analyzer. Serum magnesium (Mg) level <1.7 mg/dl was considered hypomagnesemia [18].

Statistical analysis

The data were collected from various parameters and entered in Microsoft Excel (Microsoft Excel, Redmond, WA), and analysis was done using the software package Epi Info (Version 7.1.5) from the Centers for Disease Control and Prevention (CDC, Atlanta, USA). Data were expressed as mean and standard deviation, frequency, and percentage. A chi-square test of independence was used to study the nominal variables. When there is a limitation of the chi-square test, such as its sample size requirements, we used Fisher's exact test. p-value <0.05 was considered statistically significant.

## Results

In the present study, 100 patients who presented with acute exacerbation of COPD were included. The overall mean age of the patients was 60.1±11.7 years. The mean age was higher among male patients (62.3±12.1) than female patients (57.7±11.6). Out of all patients, 27% belonged to the age group of 41 to 49 years, 25% of patients belonged to the age group of 51 to 59 years, 31% of patients from the age group 60 to 69 years, and 17% were aged more than 69 years. Among the study group, 78 (78%) patients were male while 22 (22%) were female (Table 1). Out of all patients, 87 (87%) patients had a smoking history. Among all smokers, 55% of patients had a history of smoking in the past and stopped smoking for at least six months while 32% of patients were current smokers.

**Table 1:** Age and gender-wise distribution of patients with acute exacerbation of COPD SD = standard deviation, n = number, COPD = chronic obstructive pulmonary disease

Age Interval	Male (n=78)		Female (n=22)		Total (n=100)	
	No.	Percentage	No.	Percentage	No.	Percentage
40-49 years	22	81%	5	19%	27	100%
50-59 years	18	72%	7	28%	25	100%
60-69 years	25	81%	6	19%	31	100%
>69 years	13	76%	4	24%	17	100%
Mean ± SD	62.3±12.1		57.7±11.6		60.1±11.7	

At the time of the presentation, all patients complained of breathlessness. Following that, the most common presenting symptom was cough (92%), followed by expectoration (69%), wheezing (52%), and fever (46%) (Table 2).

**Table 2:** Symptomatic presentation at the time of admission of patients with acute exacerbation of COPD (N=100) COPD = chronic obstructive pulmonary disease

Symptoms	No.	Percentage
Cough	92	92%
Expectoration	69	69%
Wheezing	52	52%
Breathlessness	100	100%
Fever	46	46%

All patients who presented with acute exacerbation of COPD underwent a chest X-ray (posteroanterior (P/A) view). The most common finding reported on the X-ray was emphysema in 41 (41%) patients. The parenchymal infiltrate was reported in 18 (18%) patients while hyperinflated lung was reported in 15 (15%) patients, lung consolidation was reported in 15 (15%) patients, and cardiomegaly was reported in 11 (11%) patients (Table 3).

**Table 3:** Profile of chest X-ray findings among patients of COPD acute exacerbation (N=100) COPD = chronic obstructive pulmonary disease

Chest X-Ray Findings	No.	Percentage
Emphysema	41	41%
Infiltrates	18	18%
Hyperinflated	15	15%
Consolidation	15	15%
Cardiomegaly	11	11%

All patients with acute COPD exacerbation have undergone electrocardiography (ECG). Out of all patients, there were no abnormalities in 21 (21%) patients. Among the remaining 79 patients, the most common ECG findings were P pulmonale plus poor R wave progression in 29%, followed by isolated P pulmonale in 18% of subjects (Table 4).

**Table 4:** Electrocardiographic (ECG) findings among patients with acute exacerbations of COPD COPD = chronic obstructive pulmonary disease

Electrocardiographic (ECG) findings	No.	Percentage
Normal	21	21%
P pulmonale	18	18%
Poor R wave progression	12	12%
P pulmonale + Poor R wave progression	29	29%
R/S ratio in lead V5 < 1	11	11%
R wave in lead V6 < 5mm	9	9%
Total	100	100%

Serum magnesium was estimated in all patients. The mean magnesium was 1.51 ± 0.4 mg/dl. Hypomagnesemia was reported in 57 patients. Among the hypomagnesemia group, 46 (80.7%) patients were admitted for more than seven days. In patients with normomagnesemia (43%), 24 (55.8%) patients were admitted for more than seven days. Compared to the normomagnesemia group, the greater number of patients with hypomagnesemia required a hospital stay of more than seven days. These findings were statistically significant (Table 5).

**Table 5:** Relation between the duration of hospital stay and serum magnesium level in patients with COPD COPD = chronic obstructive pulmonary disease P-value <0.05 considered statistically significant

Duration of hospital stay	Hypomagnesemia (n= 57)	Normomagnesemia (n= 43)	Total (n=100)
≤ 7 days	11 (19.30%)	19 (44.2%)	30
More than 7 days	46 (80.70%)	24 (55.8%)	70
Chi-square value (ꭓ2) =7.229 with 1 degrees of freedom, p-value = 0.0072			

Out of all patients with hypomagnesemia, 53 (92.9%) patients were successfully discharged while four (7.1%) were deceased. Out of all patients with normomagnesemia, 41 (95.4%) patients were successfully discharged while two (4.7%) died. A higher proportion of patients with hypomagnesemia were deceased than those with normomagnesemia. However, the difference was not statistically significant (Table 6).

**Table 6:** Relation between the outcome and serum magnesium level in patients with COPD COPD = chronic obstructive pulmonary disease P-value <0.05 is considered statistically significant.

Outcome	Hypomagnesemia (n= 57)	Normomagnesemia (n= 43)	Total (n=100)
Discharged	53 (92.9%)	41 (95.4%)	94
Deceased	4 (7.1%)	2 (4.6%)	06
Fisher's exact test, p-value=0.6971			

## Discussion

In the present study, we have included 100 patients with acute exacerbation of COPD based on our inclusion and exclusion criteria. In the present study, the overall mean age of the patients was 60.1±11.7 years, which was a higher mean age among male patients (62.3±12.1) than female patients (57.7±11.6). The proportion of the male (78%) patients was higher than the female (22%). In a similar study by Singh et al., the mean age of patients was 60.4±6.5 years, and the proportion of male and female patients was 58% and 42%, respectively [19]. A study by Bhatt et al. reported a higher mean age of 71.9±10.9 years; the male and female patients were 43% and 57%, respectively [20]. In a study conducted by Shah et al., the mean age of the patients was 62.5 years, and the proportion of male (58.4%) patients was higher than the female (41.6%) patients [21]. So, similar studies of acute exacerbation of COPD had reported a wide variation in demographic variables like age and gender of patients.

Out of all patients, 87% of patients had a smoking history. Among all smokers, 55% of patients had a history of smoking in the past and stopped smoking for at least the last six months while 32% of patients were current smokers. In a study conducted by Kanimozhi et al. to study the relation between serum magnesium and acute exacerbation, the proportion of non-smokers is almost similar (12%) [22]. In a survey conducted by Bhatt et al., among acute COPD patients, 90% of patients were either ex-smokers or current smokers [20].

At the time of the presentation, all patients had complained of breathlessness. Following that, the most common presenting symptom was cough (92%), followed by expectoration (69%), wheezing (52%), and fever (46%). In a study by Shah et al., the most common presenting symptoms were dyspnea (100%), followed by cough and expectorations in 96% and 63%, respectively. In a study by Singh et al., dyspnoea was the most common symptom following cough and expectorant at the time of admission [19]. The high prevalence of dyspnoea and cough showed that most patients were very severe at presentation.

The most common finding reported on chest X-rays was emphysema in 41% of patients, followed by parenchymal infiltrate (18%), hyperinflated lung (15%), lung consolidation (15%), and cardiomegaly (11%). In a study done by Singh et a., the most common X-ray findings were emphysema followed by parenchymal infiltrates in 47.05% and 17.64%, respectively [19]. In our study, 79% of patients had an abnormal ECG. The most common ECG changes were P pulmonale plus poor R wave progression in 29% and isolated P pulmonale in 18%. The association of prolonged P wave dispersion and right atrial enlargement (RAE) with COPD may explain the increased prevalence of atrial arrhythmias [23-24].

In the present study, hypomagnesemia was reported in 57% of patients with acute exacerbation of COPD. In some other studies by Singh et al. [19] and Kumar et al. [25], hypomagnesemia was reported in 34% and 45% of acute COPD patients. A wide variation is registered in the prevalence of hypomagnesemia among patients with acute exacerbation of COPD in different literature. In a study by Shah et al., hypomagnesemia was reported in 33.8% and the mean serum magnesium level was 1.9±0.7 mg/dl [21]. Our study’s mean serum magnesium level was 1.51 ± 0.4 mg/dl. In a survey conducted by Kanimozhi et al., the mean serum magnesium was slightly higher (1.6±0.3) than in the present study [22].

Out of all patients with hypomagnesemia, 80.7% were admitted for more than seven days while in the normomagnesemia group, 55.8% were admitted for more than seven days. Hospital stay among hypomagnesemia patients was significantly higher than that among normomagnesemia patients in our study. The findings observed were statistically significant (p-value 0.007). In a study conducted by Singh et al., hospital stays longer than seven days were also reported to be higher in the hypomagnesemia group (64.70%) than in the normomagnesemia group (57.75%) [19]. The potential factors associated with the more extended hospital stay might be the requirement of ventilator care studied by Groenewegen et al. [26] and Roberts et al. [27].

The overall mortality reported in the present study was 6%. The mortality rate was higher in the hypomagnesemia group (7.1%) than in the normomagnesemia group (4.7%), but the difference was not statistically significant (p-value 0.622). In a survey conducted by Bhatt et al., the mortality rate in acute exacerbation of COPD was 5% [20]. Some other studies were undertaken by Gaude et al. [28] and Wang et al. [29], which reported an outcome of 12% and 9.9% among hospitalized patients with acute exacerbation of COPD.

Magnesium sulfate has been used to treat severe acute exacerbations of asthma not responding to bronchodilators or steroids via nebulization or intravenous injection [30]. Magnesium mainly affects bronchial smooth muscles and acts as a bronchodilator by inhibiting voltage-dependent calcium channels [31]. Asthma and COPD both have obstructive pathology and comparable line of management in acute exacerbation episodes. So, the role of serum magnesium in COPD exacerbations can be studied to improve outcomes [32].

In a study by Zanforlini et al., treating COPD patients with oral magnesium had shown an anti-inflammatory role. Still, it does not substantially improve lung function, physical activity, and quality of life in COPD patients [33]. A recent systematic review and meta-analysis study by Jahangir et al. has studied that intravenous magnesium was associated with favorable deviation of FEV1 and peak expiratory flow rate (PEFR). It has decreased residual volume and impacts in reducing odds of admission in patients with acute exacerbations of COPD. It is suggested that magnesium sulfate can be used as adjunctive therapy in treating acute exacerbations of COPD patients [32].

This study has its limitations. For acute exacerbation of COPD, frequent hospital readmissions and their associated factors were not considered. The baseline magnesium level before an acute exacerbation episode was unavailable in our patients. Further studies with a larger sample size and a more extended follow-up period are required to validate the results.

## Conclusions

Hypomagnesaemia is a common finding in acute exacerbation of COPD. The level of magnesium found is related to the length of hospital stay, but it is not related to mortality among patients with acute exacerbation of COPD. Treatment with magnesium to correct hypomagnesemia in acute exacerbation of COPD can be helpful for a better outcome. Looking forward, a multicentric longitudinal study on the relation of magnesium with the management and prognosis of COPD is needed.
